# Construction of a hospital intelligent integrity supervision platform: digital and intelligent practices for enhancing ethical governance in healthcare

**DOI:** 10.3389/fpubh.2026.1769451

**Published:** 2026-02-20

**Authors:** Xiaofei Chen

**Affiliations:** Discipline Inspection and Supervision Office, Hangzhou Xixi Hospital, Hangzhou, Zhejiang, China

**Keywords:** compliance risk management, data integration, digital governance, ethical healthcare, healthcare integrity, intelligent supervision platform, smart hospital

## Abstract

**Introduction:**

The digital transformation of governance in public hospitals is crucial for enhancing integrity and compliance. This study investigates the design, implementation, and impact of an Intelligent Integrity Supervision Platform in a hospital setting, aiming to provide a replicable model for data-driven ethical governance.

**Methods:**

A case study approach was employed at Hangzhou Xixi Hospital, combining system analysis with outcome evaluation. The platform integrated multi-source data and established real-time early-warning mechanisms. Quantitative data on integrity incidents, medical disputes, and patient satisfaction were collected from quarterly reports (2024-2025) and analyzed to compare pre- and post-implementation periods.

**Results:**

Following the platform's launch, integrity risk incidents decreased stepwise, achieving a 100% reduction (zero incidents) in the second half of 2025 compared to the baseline. Medical disputes fell by 75% year-on-year. Patient satisfaction metrics improved, including an increase in the platform complaint resolution rate from 95.1% to 97.3% and a 19.7% decline in discharge follow-up issue rates.

**Discussion:**

The platform demonstrates the efficacy of digital-intelligent systems in transforming ethical governance by enabling proactive risk prevention, standardizing operations, and fostering accountability. It aligns with global efforts to leverage technology for anti-corruption in healthcare. The study concludes that such platforms serve as strategic tools for systemic governance reform, contributing to both institutional integrity and patient safety.

## 1 Introduction

In the era of digital transformation, hospitals are increasingly adopting advanced technologies such as artificial intelligence (AI), big data, the Internet of Things (IoT), and cloud computing to improve service quality and operational efficiency (1). Within this context, ethical governance has emerged as a critical component of healthcare reform, directly impacting patient safety, institutional credibility, and public trust. The integration of digital tools into governance frameworks presents new opportunities to enhance transparency, standardize decision-making, and prevent misconduct (2).

Big data–enabled supervision, characterized by closed-loop processes of data collection, intelligent analysis, and dynamic warning, has become a cornerstone of modern compliance oversight (3). In healthcare, such systems allow for real-time monitoring of high-risk areas such as procurement, prescription practices, and clinical conduct. For instance, regional health authorities in China have begun implementing “Health Brain” platforms to enable granular oversight of medical indicators, forming a smart supervision model capable of real-time data capture and risk alerting (4).

Against this backdrop, Hangzhou Xixi Hospital developed and deployed an Intelligent Integrity Supervision Platform to achieve precise, process-oriented supervision of key operations, personnel, and workflows. By integrating multi-source data and establishing an early-warning mechanism, the platform facilitates a closed-loop management cycle of “data collection → intelligent analysis → dynamic warning → intervention.” Grounded in the core principles of systemic defense in patient safety—which advocates for designing organizational systems that reduce reliance on individual vigilance by embedding safety, integrity, and accountability into daily workflows—this study examines the platform not merely as a technological tool but as a structural intervention for proactive governance (5). We posit that by integrating multi-source data and establishing closed-loop early-warning mechanisms, the platform operationalizes systemic defense, shifting the governance paradigm from reactive punishment to proactive prevention and supportive oversight. Consequently, this paper details the platform's design, implementation, and outcomes within this theoretical framework, providing evidence for its effectiveness and offering a replicable model for digital governance initiatives in healthcare institutions worldwide.

## 2 Background

### 2.1 Policy context

National and regional health policies have increasingly emphasized the role of informatization in promoting integrity and compliance within public hospitals. The Large Hospital Inspection Work Plan (2023–2026) issued by China's National Health Commission mandates the establishment of risk prevention systems through digital means. In 2021, the Zhejiang Provincial Health Commission released the Evaluation and Management Measures for Clean Construction Index in Public Hospitals (Trial), providing a structured framework for assessing and monitoring ethical governance (6). More recently, the 2025 Work Priorities for Rectifying Unethical Practices in Medical Procurement and Services explicitly calls for “penetrative audit supervision” and end-to-end traceability of pharmaceuticals and medical consumables (7), reinforcing the need for integrated, intelligence-driven oversight.

### 2.2 Practical necessity

Healthcare remains a high-risk sector for integrity violations due to complex operational environments, fragmented data systems, and traditional oversight limitations. Common challenges include data silos, delayed risk detection, and insufficient analytical capacity (8). Traditional manual supervision is inadequate for processing large-scale operational data in real time, creating an urgent need for intelligent systems that enable proactive risk management. The proposed platform addresses these gaps by offering dynamic monitoring, automated alerts, and data-driven decision support, thereby enhancing the precision and efficiency of compliance oversight (9).

## 3 Methods

This study employs a case study approach combined with system analysis and outcome evaluation. The Intelligent Integrity Supervision Platform at Hangzhou Xixi Hospital serves as the primary case. Data were collected from quarterly compliance reports (2024–2025), red envelope return logs, patient satisfaction surveys, and internal dispute records. Functional modules were designed based on hospital operational workflows and regulatory requirements. Quantitative metrics were analyzed to assess changes in compliance incidents, satisfaction rates, and administrative efficiency before and after platform deployment.

### 3.1 Study limitations

This study employs a pre-post implementation comparison within a single institution. While the observed temporal associations are compelling, we acknowledge that factors such as concurrent national anti-corruption campaigns, institutional reforms, or leadership changes could have contributed to the outcomes. Therefore, the findings primarily demonstrate association rather than definitive causation. We further acknowledge three key limitations: First, the single-hospital design limits generalizability to diverse healthcare systems, institutional cultures, or regulatory contexts. Second, the absence of a control group prevents definitive attribution of outcomes solely to the platform, as concurrent regional health digital reform policies and national ethical governance initiatives may have synergistic effects. Third, the relatively short follow-up period (18 months) limits conclusions on long-term sustainability. Future multi-center studies with controlled designs and longitudinal follow-up (≥3 years) are recommended to strengthen causal inference and scalability. Additionally, the evaluation period (2024–2025) is relatively short for assessing long-term sustainability; plans for ongoing monitoring are in place.

## 4 Platform design and components

### 4.1 Architectural overview

**Figure 1:**
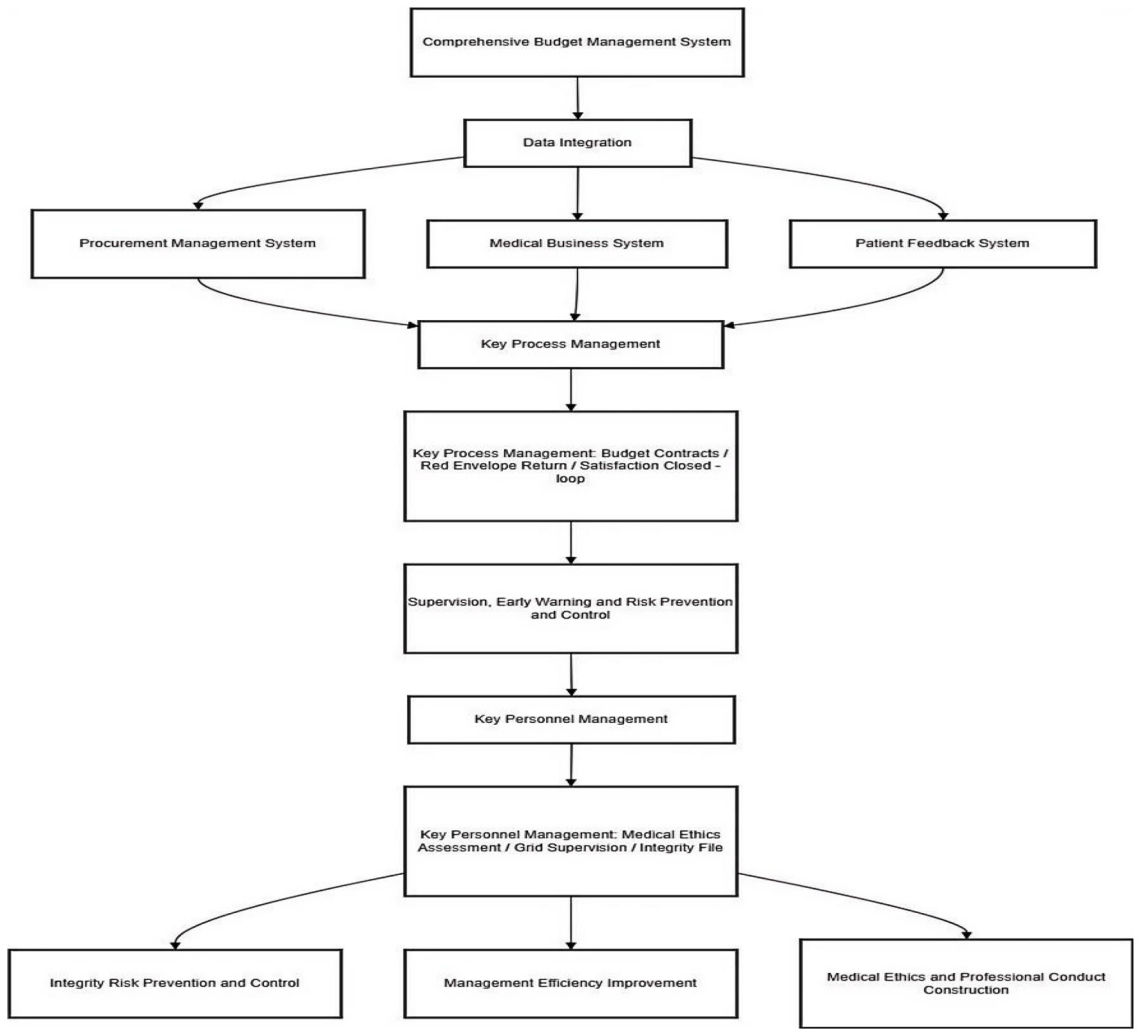
Architecture of the hospital intelligent integrity supervision platform.

The platform is structured around four core modules (Figure 1):

1. Data Collection and Integration Module – interfaces with hospital information systems (HIS, ERP, procurement, finance) to create a unified data repository.
2. Key Process Management Module – oversees budget-contract cycles, red envelope returns, satisfaction tracking, and issue accountability.
3. Key Personnel Management Module – manages ethics assessments, grid-based supervision, integrity archives, and daily compliance tasks.
4. Supervision and Early-Warning Module – monitors high-risk areas (procurement, rotations, red envelopes) and triggers real-time alerts for anomalies.

### 4.2 Data integration and governance

The platform integrates over 30 key performance and compliance indicators from multiple hospital subsystems. Using ETL (Extract, Transform, Load) pipelines for data integration and warehousing, heterogeneous data are cleansed, standardized, and stored in a structured data pool (10). To ensure security, compliance, and accountability, the platform implements a three-tier governance framework: (1) Data permissions: Role-based access control (RBAC) strictly limits sensitive data access (e.g., personal integrity files, individual prescribing data) to authorized staff in the Ethics and Compliance Office, with dynamic data masking for patient identifiers (11); (2) Privacy protection: A “data sandbox” is used for analytical queries, where raw data are desensitized (e.g., tokenization of patient IDs, aggregation of department-level metrics) in compliance with HIPAA (12) and China's Medical Data Security Management Measures (13); (3) Accountability mechanisms: The hospital's Ethics Committee conducts quarterly audits of platform operations, including data access logs and early-warning handling records, with annual performance reviews by a third-party professional institution (14). The Hangzhou Municipal Health Commission retrieves and aggregates relevant data through the platform interface, and accesses early-warning information and operational reports to ensure regulatory alignment.

### 4.3 Process management

- Budget and Contract Supervision: Tenders, contract execution, and payment milestones are tracked in real time.
- Red Envelope Management: Enables self-reporting and automated ethics scoring.
- Patient Satisfaction System: QR-based feedback collection and closed-loop complaint resolution.
- Issue Accountability: Assigns, tracks, and evaluates corrective actions for compliance violations.

### 4.4 Personnel supervision

- Ethics Assessment: Online applications, automated scoring, and batch approval.
- Grid Supervision: Multi-level reporting for full coverage of staff conduct (15).
- Integrity Archives: Digital declarations of assets, external engagements, and conflicts of interest.
- Daily Compliance Tools: Online applications for encrypted devices, integrity pledges, and decryption services.

### 4.5 Early-warning and risk control

- Procurement Monitoring: Alerts for unqualified suppliers, price deviations, and procedural breaches (16).
- Rotation Reminders: Automated notifications for personnel due for rotation.
- Misconduct Alerts: Instant escalation of patient complaints regarding red envelopes or rebates (17).

## 5 Results

### 5.1 Reduction in integrity incidents

Following the platform's launch in July 2024, integrity risk incidents decreased from 5 in the first half of 2024 (baseline) to 2 in the second half of 2024, representing a reduction of 60% (Rate Ratio, IRR = 0.40; 95% CI: 0.08–2.09). The downward trend continued in the first half of 2025, with only one incident recorded—a further 50% reduction compared to the initial post-implementation period (IRR = 0.50; 95% CI: 0.05–5.57). Most notably, during the second half of 2025, no integrity risk incidents were recorded, achieving a 100% reduction compared to the baseline and resulting in an rate of zero (95% CI: 0.0–3.7 per half-year). Although the confidence intervals are wide due to the low absolute number of events—a reflection of the hospital's overall low baseline risk—the consistent, stepwise reduction culminating in zero incidents over 18 months provides strong evidence of the platform's sustained, real-world effectiveness in risk prevention (Table 1).

**Table 1:** Integrity risk incidents before and after platform implementation.

**Period**	**Integrity risk incidents**	**Change (vs. previous period)**	**Incidence rate per half-year (95% CI)**	**Notes**
1H 2024 (Baseline)	5	–	5.0 (1.6–11.7)	Pre-implementation
2H 2024 (Initial Post-implementation)	2	−60%	2.0 (0.2–7.2)	Platform launched in July 2024
1H 2025 (Sustained Operation)	1	−50%	1.0 (0.03–5.6)	Trend sustained
2H 2025 (Long-term Operation)	0	−100%	0.0 (0.0–3.7)^*^	Achieved zero incidents

^*^Confidence intervals (CI) for Poisson-distributed count data were calculated using the exact method (Garwood method). The upper limit of the CI for zero events is 3.7. The platform's launch marks the transition from baseline to intervention phases.

### 5.2 Decrease in medical disputes

Medical disputes fell from 4 cases in 2024 to 1 case in 2025, a 75% year-on-year reduction (Table 2), reflecting improved procedural standardization and communication transparency.

**Table 2:** Medical disputes before and after platform implementation.

**Year**	**Disputes**	**Change**
2024	4	–
2025	1	−75%

### 5.3 Improved Patient Satisfaction

Patient satisfaction indicators showed consistent improvement post-implementation (Table 3). The platform complaint resolution rate increased from 95.1% to 97.3%, while discharge follow-up issue rates declined by 19.7%.

**Table 3:** Patient feedback metrics before and after implementation.

**Metric**	**Pre-launch**	**Post-launch**	**Change**
Government hotline complaint resolution	100%	100%	–
Platform complaint resolution	95.1%	97.3%	+2.2%
Discharge follow-up issue rate	13.89%	11.15%	−19.7%
Outpatient SMS issue rate	0.26%	0.24%	−7.7%

## 6 Discussion

The Intelligent Integrity Supervision Platform exemplifies how digital-intelligent systems can transform ethical governance in healthcare (18). By enabling real-time data integration, automated monitoring, and proactive risk alerts, the platform enhances institutional transparency, reduces misconduct, and fosters a culture of accountability. These outcomes align with international efforts to leverage technology for anti-corruption and governance improvement in public health systems (19).

Beyond compliance and satisfaction improvements, the platform indirectly enhances patient safety by standardizing high-risk processes. For example, real-time monitoring of procurement reduces the risk of substandard medical consumables entering clinical practice, while standardized doctor-patient communication (facilitated by the satisfaction closed-loop system) minimizes misunderstandings that may lead to medical disputes. This alignment between ethical governance and patient safety underscores the platform's value as a systemic intervention for healthcare quality improvement (20).

### 6.1 Balancing supervision with psychological safety: fostering a just culture

A critical consideration in implementing an integrity supervision platform is its potential impact on staff psychological safety and the organizational culture (21). To avoid creating an atmosphere of punitive surveillance, our platform was designed with principles of supportive governance and a just culture in mind. Thus, the platform redefines traditional supervision as a form of enabling governance and systemic support, aiming to prevent errors rather than merely punish them.

1. Differentiation between Error and Misconduct: The early-warning system is calibrated to flag patterns indicative of potential misconduct (e.g., consistent deviations from procurement guidelines), not single, unintentional human errors. All alerts undergo human review by the Ethics and Compliance Office before any action is taken, ensuring context is considered.
2. Feedback and Learning: When anomalies are identified, the primary response is not punitive but investigative and educational. Supervisors use the data as a basis for constructive feedback and, if necessary, systemic process improvement (e.g., clarifying ambiguous procurement rules).
3. Safeguards against Punitive Use: Access to individualized monitoring data is strictly limited via RBAC. The platform's official use is framed to staff as a “systemic defense tool” to protect both the institution and individuals from integrity risks, aligning with the goal of creating a “learning health system” (22). This approach aims to move beyond mere monitoring toward enabling governance, where transparency supports accountability while preserving trust and the willingness to report near-misses—a cornerstone of high-reliability organizations.

The case of Hangzhou Xixi Hospital demonstrates that such platforms are not merely technological upgrades but strategic tools for systemic governance reform. Future developments may incorporate machine learning for predictive analytics, potentially linking integrity monitoring with direct clinical safety indicators (e.g., medication error patterns, diagnostic delay alerts) to further bridge ethical governance and patient outcomes, blockchain for tamper-proof audit trails, and interoperable designs for multi-institutional data sharing.

## 7 Conclusion

This study presents the design, implementation, and impact of an Intelligent Integrity Supervision Platform in a public hospital setting. Results confirm its effectiveness in reducing compliance risks, standardizing operations, and improving patient satisfaction. The platform offers a replicable model for healthcare institutions seeking to strengthen ethical governance through digital means. To sustain the platform's effectiveness, Hangzhou Xixi Hospital has implemented continuous staff training (quarterly workshops on ethical data use and platform operation) and regular system audits (monthly operational reports, annual third-party evaluations). Future efforts will focus on multi-center replication to verify scalability, integrating staff behavioral metrics and cost–benefit analysis to provide a more holistic evaluation, and extending longitudinal follow-up to strengthen causal inference. Ongoing refinement of data security, algorithmic accuracy, and governance integration will further advance the role of intelligent systems in promoting integrity and trust in healthcare.

## Data Availability

The original contributions presented in the study are included in the article/supplementary material, further inquiries can be directed to the corresponding author.
